# Evaluation of Facial Artery Course Variations, Diameters, and Depth Using Doppler Ultrasonography: A Systematic Review and Meta‐Analysis

**DOI:** 10.1111/jocd.70431

**Published:** 2025-08-28

**Authors:** Mohammad Reza Pourani, Mandana Ebrahimzade, Ehsan Goudarzi, Martin Kassir, Reza Robati, Hamideh Moravvej, Fahimeh Abdollahimajd

**Affiliations:** ^1^ Skin Research Center Shahid Beheshti University of Medical Sciences Tehran Iran; ^2^ Department of Dermatology Shohada‐e Tajrish Hospital, Shahid Beheshti University of Medical Sciences Tehran Iran; ^3^ School of Medicine Shahid Beheshti University of Medical Sciences Tehran Iran; ^4^ Worldwide Laser Institute Dallas Texas USA; ^5^ Research Center of Artificial Intelligence in Health Shohada‐e Tajrish Hospital, Shahid Beheshti University of Medical Sciences Tehran Iran; ^6^ Clinical Research Development Unit Shohada‐e Tajrish Hospital, Shahid Beheshti University of Medical Sciences Tehran Iran

**Keywords:** anatomic variation, color doppler ultrasonography, diagnostic imaging, facial artery, systematic review, vascular injury

## Abstract

**Background:**

The facial artery (FA), as the primary vascular structure of the face, exhibits numerous anatomical variations. Determining its anatomical variation (vascular mapping) by ultrasonography (US)—a non‐invasive, low‐cost, practical, and bedside‐applicable imaging technique—helps achieve safe aesthetic injections, averting complications such as ischemia and necrosis.

**Objectives:**

This study aimed to evaluate the anatomical characteristics of FA under the guidance of US, including course variations in relation to the nasolabial fold (NLF), depth, and diameter.

**Methods:**

This systematic review and meta‐analysis used the 2020 update of the PRISMA guidelines. The detailed protocol is registered with PROSPERO. Original studies with extractable numerical data that applied Doppler US in normal adult populations to illustrate FA anatomy were evaluated. The primary two outcomes of the study were (1) the pooled visualization rate of the FA in each of the three anatomical levels and (2) the pooled prevalence of each FA course variation according to the NLF.

**Results:**

A systematic search through the three online databases identified 1087 records, of which 580 underwent title and abstract screening after duplicate removal, and 10 studies were included. The visualization rates at three anatomical facial levels were 100%, 99.9%, and 99.8% of all arteries. FA running medial to the NLF (type A) (46.2%), followed by FA crossing medial to lateral of the NLF (type C) (22.5%), were the most frequent courses of FA in relation to the NLF. In contrast, the FA running lateral to the NLF (type B) (12.0%) was the scarcest variation. The FA became more profound in the skin layers as it ascended in the face from level 1 (6.27 mm) to level 3 (8.04 mm). Moreover, the FA narrowed in diameter from level 1 (2.14 mm) to level 3 (1.46 mm) as it branched out and approached its termination point. Therefore, the total prevalence of angular, lateral nasal, and superior labial artery as the final branch of the FA in our study was 71.8%, 27.9%, and 5.7%, respectively.

**Conclusions:**

According to the results, the US could detect FA in almost all cases at the three levels of the face, including the lower border of the mandible, cheilion, and lateral nasal ala. This systematic review and meta‐analysis provided a comprehensive anatomical knowledge of the Doppler‐visualized FAs as an exemplary schema of pre‐procedural vascular mapping that can help aestheticians prevent intravascular injections, exerting safer performance during cosmetic procedures.

**Trial Registration:**

PROSPERO number: CRD42024616195

## 1 Introduction

In recent years, minimally invasive cosmetic procedures, such as soft‐tissue filler injections, have gained immense popularity for restoring senile facial changes, including deep wrinkles and prominent nasolabial folds (NLF), due to ongoing age‐related tissue loss [1, 2]. Although filler augmentations are generally considered safe, the recent global surge in their application has raised concerns about ischemic complications, such as soft tissue necrosis, ophthalmoplegia/double vision, unilateral or bilateral visual loss, and ischemic strokes resulting from vascular occlusion due to filler embolization or vascular compression [1, 3, 4]. According to Ozturk et al. [5], the nose area (33.3%) and NLF (31.2%) were the most frequently affected regions for necrosis, while almost half of the blindness cases were preceded by glabellar injections. Despite the low incidence rate (0.0001%) [5], of severe complications, the uncertain reversibility of these ischemic adverse events necessitates meticulous consideration of facial vasculature to reduce arterial invasion [3, 6].

The facial artery (FA), as the leading vascular structure of the face, exhibits numerous anatomical variations. The FA mainly enters the face below the pre‐masseteric notch, curves around the lower border of the mandible, and runs obliquely from the anteroinferior border of the masseter muscle toward the medial canthus [7]. Through this oblique portion of the artery, labile branches (superior/inferior) sprout near the cheilion, and the remaining arterial trunk ascends near the NLFs. The FA gives off the lateral nasal artery (LNA) adjacent to the nasal sidewall and continues as an angular artery (AA) to anastomose with the dorsal nasal artery (DNA), a terminal branch of the ophthalmic artery [8, 9]. Acknowledging its anatomical variation is helpful for safe aesthetic injections and also orthognathic and reconstructive flap surgeries, reducing iatrogenic complications [10].

Ultrasonography offers a non‐invasive, low‐cost, practical, and bedside‐applicable imaging technique used as a diagnostic modality as well as a therapeutic procedure. In several fields of medicine, including emergency medicine, anesthesiology, orthopedics, dermatology, and internal medicine, many interventions are guided by the US, taking advantage of its real‐time interactive demonstrations [11, 12].

Dermatologic ultrasound (US) applications have gained significant interest in various diagnostic, therapeutic, and cosmetic fields, setting a new benchmark in the visualization of cutaneous and subcutaneous layers and their three‐dimensional (3D) anatomical relationships, including the trajectories of blood vessels. For dermatologic applications, linear probes are required to evaluate skin layers, providing an illustration of superficial structures [13, 14, 15]. The role of dermatologic high‐ and ultra‐high frequency US in infections, inflammatory dermatoses (e.g., psoriasis [16], lichen planus, pemphigus vulgaris), metabolic and genetic disorders, specific cutaneous structure skin disorders, vascular and external‐agent‐associated disorders, neoplastic diseases and tumor margin mapping [17, 18], and finally, aesthetics has been reviewed comprehensively in previous literature [14]. High‐frequency US has sparked a revolution in aesthetics by providing real‐time visualization of the patient's anatomy, needle or cannula tip, and previously deposited fillers [19] with high diagnostic accuracy [14]. Doppler mode is always recommended to delineate the vascular structures of the interested region and their positioning in relation to the cannula or needle [20], helping to prevent vascular invasions and thereby devastating ischemic complications. Hence, Doppler high‐frequency US is being advocated as the first‐line imaging modality in the aesthetic field [15, 21, 22]. The Doppler US allows professionals to establish safe injection planning in live individuals by preoperative neurovascular mapping with appreciation of anatomical variations and identification of danger zones [22]. Moreover, Doppler US enables more efficient treatment of filler complications by localization of the errant filler material [19] and guiding the hyaluronidase administration process [23]. However, despite the global trend toward integration of Doppler US into cosmetic procedures, there is an apparent gap for Doppler US to be a part of routine standard‐of‐care in aestheticians' daily practice [13, 21]. Further research with stronger evidence is needed to validate current knowledge in this field and enhance the role of Doppler US in cosmetics by thoroughly weighing its benefits against its limitations and challenges.

In this systematic review and meta‐analysis, we aimed to validate the sufficient efficacy of Doppler US in visualizing FA at different facial levels. Furthermore, FA anatomical characteristics under the guidance of US, including course variations in relation to the NLFs, depth, diameter, terminal branch, and detection rate of each branch, were recapitulated to provide practical information for aesthetic injectors. Understanding the applicability and validity of Doppler ultrasonography in delineating FA's anatomy leads to decreased possible dangers associated with procedures carried out in FA territory.

## 2 Methods

This systematic review and meta‐analysis was performed and presented following the 2020 update of Preferred Reporting Items for Systematic Reviews and Meta‐Analyses (PRISMA) guidelines. The detailed protocol is registered with the International Prospective Register of Systematic Reviews (PROSPERO) (Registration ID: CRD42024616195).

### 2.1 Eligibility Criteria and Search Strategy

On September 26th, 2024, the three online databases of MEDLINE, Scopus, and Embase were thoroughly searched by M.E. for all articles published until September 2024 according to a pre‐established structured search strategy (available in the Data S1). No language restrictions or other filters allowed the detection of the maximum number of records concerning the facial artery anatomy and variations. The obtained citations were screened meticulously by title/abstract review to identify studies that exclusively used Doppler US to determine the anatomy of the facial artery. The snowball citation tracking was conducted by M.E. on October 15th, 2024, by investigating the references of the included studies after full‐text screening, relevant articles, and reviews to detect any potential records for inclusion.

The full text of original studies with extractable numerical data that applied Doppler US in normal adult populations of any nationality to illustrate facial artery anatomy were assessed. Studies that provided data by Doppler US regarding at least one of the following items were included and further entered into the synthesis process: (1) the number of visualized facial arteries in any of the three anatomical landmarks of the lower border of the mandible, the cheilion, and the lateral nasal ala; (2) the relationship between the FA and the nasolabial fold (NLF). The exclusion criteria were as follows: (1) case reports, case series, reviews, and letters to the editors; (2) studies comprised of patients with any facial vascular malformation, pathology, or history of head and neck surgery distorting the FA anatomy; (3) studies with anatomical data obtained from cadaveric dissection, angiography, computed tomography (CT), or any modalities other than Doppler US; (4) studies investigating anatomical characteristics of other facial vessels; and (5) studies with incompatible data to our classifications or no relevant quantitative or extractable data.

### 2.2 Selection Process, Data Collection, and Extraction

After a comprehensive search through the three online databases mentioned, the obtained records were title/abstract screened by two independent reviewers (M.E. and M.P.), and merely the Doppler US‐applied relevant articles were sought for retrieval. The same reviewers independently assessed the retrieved full texts for eligibility by applying the inclusion and exclusion criteria. Any disagreements in each step were resolved via consultation and, if necessary, by a third expert (F.A.). Two independent reviewers (M.E. and E.G.) conducted the data extraction process using a standardized data collection form designed according to this study's requirements in Microsoft Excel. A senior reviewer (M.P.) judged the conflicts in the data extraction process after a consensus discussion. The following data were collected: Baseline characteristics (first author's name, year, continent, country, number of patients and hemi faces, number of males and females, age range, mean age (standard deviation, SD)), body mass index (BMI) (kg/m^2^), and the FA anatomical data reported by individual studies in both right and left hemi faces including the number of visualized FAs, mean depth and diameter in each anatomical landmark, number of each FA course variation according to the NLF, and each FA termination pattern. The anatomical data regarding the FA branches (AA, LNA, or superior/inferior labile artery (SLA/ILA)) were also extracted if reported in any of the included studies. Data reported in the subsets of males and females were extracted separately for each gender.

### 2.3 Outcome Measures

There were two outcomes defined as “co‐primary outcomes” in this systematic review and meta‐analysis: (1) The pooled visualization rate of the FA in each of the three anatomical landmarks of the lower border of the mandible, the cheek, and the lateral nasal ala; (2) The pooled prevalence of each FA course variation (medial, lateral, crossing from medial to lateral, crossing from lateral to medial) according to the NLF. The co‐primary outcomes were expressed as percentages with their corresponding 95% confidence intervals (95% CIs).

**FIGURE 1:**
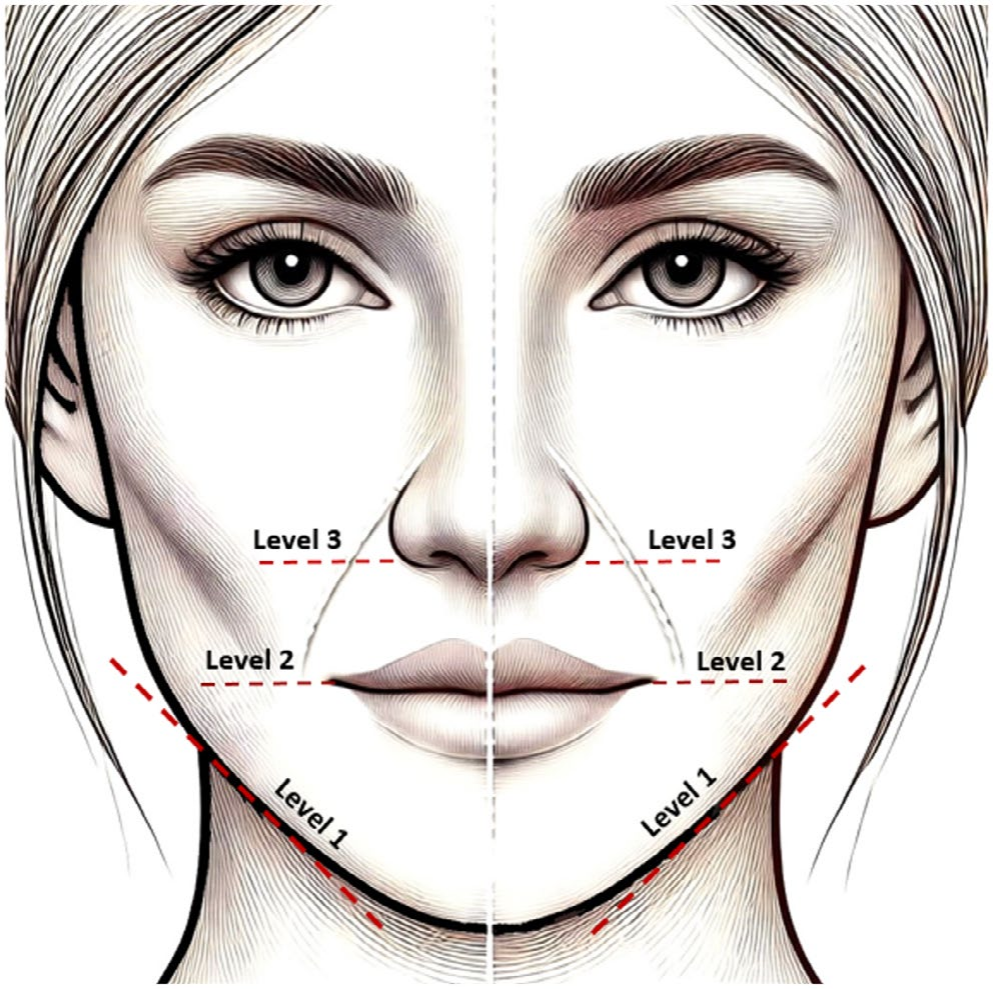
Three anatomical landmarks, Level 1: The lower border of the mandible, Level 2: Cheilion, Level 3: Lateral nasal ala.

**FIGURE 2:**
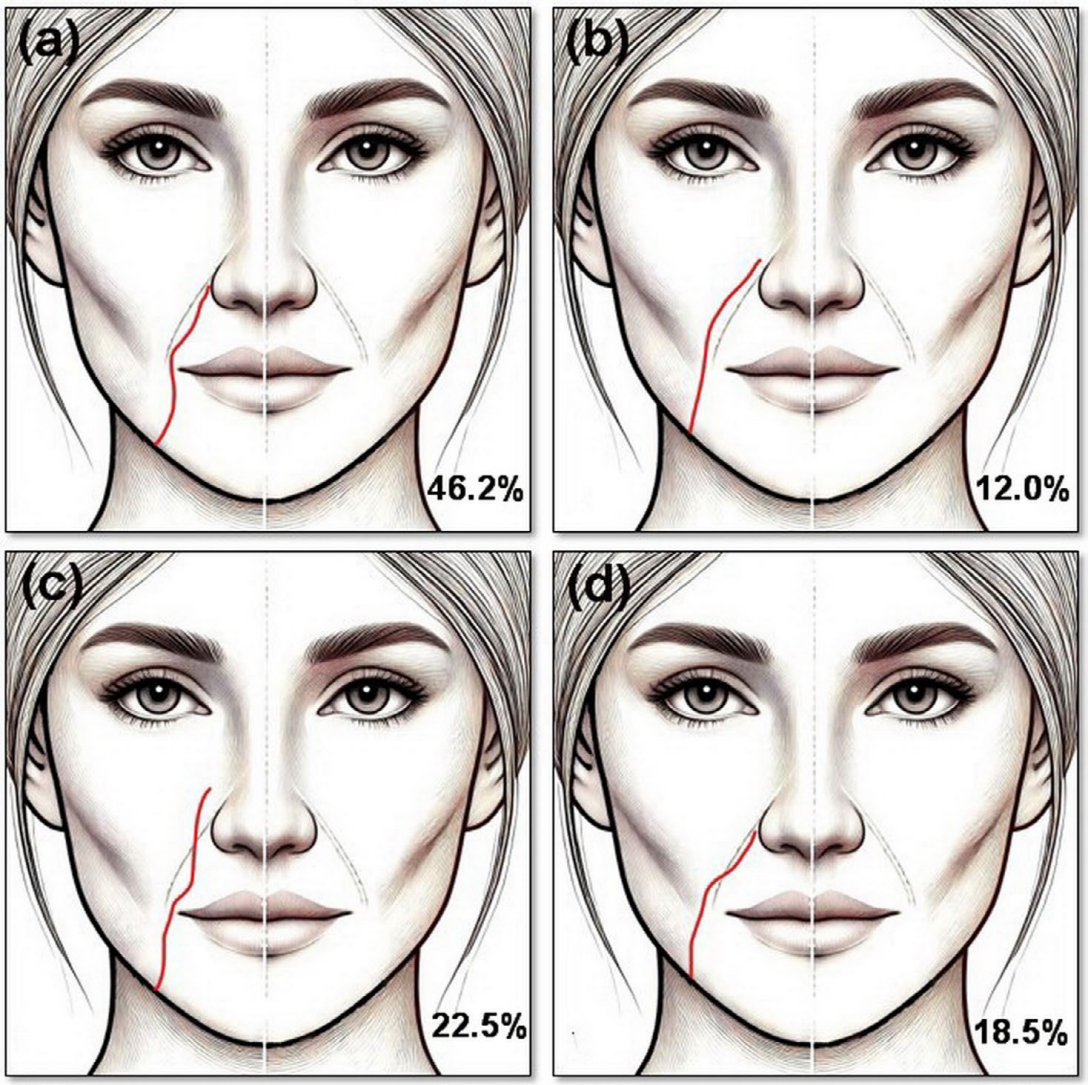
Facial artery course variations according to the nasolabial fold. (a) Type A: FA is parallel and medial (inferior) to the NLF. (b) Type B: FA is parallel and lateral (superior) to the NLF. (c) Type C: FA crosses the NLF from medial (inferior) to lateral (superior). (d) Type D: FA crosses the NLF from lateral (superior) to medial (inferior).

The three levels of the lower border of the mandible (level 1), cheilion (level 2), and lateral nasal ala (level 3) were selected as the accepted landmarks for the evaluation of FA according to the existing literature (Figure 1). The relationship between the FA and the NLF was classified into four types based on the classification system first established by Ten et al. [24], Type A: FA is parallel and medial (inferior) to the NLF. Type B: FA is parallel and lateral (superior) to the NLF. Type C: FA crosses the NLF from medial (inferior) to lateral (superior). Type D: FA crosses the NLF from lateral (superior) to medial (inferior) (Figure 2).

Secondary outcomes were as follows: (1) the pooled mean depth of the facial artery in each of the three anatomical landmarks of the lower border of the mandible, cheek, and lateral nasal ala; (2) the pooled mean diameter of the facial artery in each of the three anatomical landmarks of the lower border of the mandible, cheilion, and lateral nasal ala (these two secondary outcomes were measured in millimeters (mm) and are presented as pooled mean values with 95% CIs); (3) the pooled mean difference (MD) (mm) and pooled standardized mean difference (SMD) (mm) of the depth and diameter differences between right and left hemi faces with their corresponding 95% CIs (right vs. left MD and SMD). The pooled SMD values of 0.2–0.5, 0.5–0.8, and > 0.8 were interpreted as small, medium, and large, respectively [25].

Tertiary outcomes were (1) the pooled prevalence (%) with 95% CI for each termination pattern of the FA, presented by its final branch (AA, LNA, or SLA), and (2) the pooled visualization rate (%) with 95% CI of each FA branch (AA, LNA, SLA, or ILA) for which data regarding its detection rate were available from the included studies.

All outcomes were measured for both right and left hemi faces as well as the total values. Symmetry was only evaluated in the “FA course variation according to the NLF” outcome since it was the only outcome with sufficient collected data.

### 2.4 Risk of Bias Assessment

The risk of bias (quality) assessment was performed by application of the Anatomical Quality Assessment (AQUA) tool comprised of a total of 25 questions in five domains of aim and subject characteristics (Domain 1), study design (Domain 2), methodology characterization (Domain 3), descriptive anatomy (Domain 4), and reporting of results (Domain 5) [26]. This assessment was carried out independently by two reviewers (M.E. and M.P.), and discrepancies were decided by a third expert (F.A.). A detailed list of the AQUA questions can be found in the caption of Table 3.

### 2.5 Statistical Analysis

STATA 17.0 statistical software was utilized by E.G. to conduct all analyses. Categorical variables were extracted as frequencies (prevalence %) and continuous variables as mean (SD). The individual studies' mean depth and diameter data were entered into the synthesis process only if the number of visualized FAs was available for that specific anatomical level to provide a precise sample size for further calculations. Besides the defined outcomes in Section 2.3, the pooled mean diameter of the facial artery at level 1 (the lower border of the mandible) was also calculated separately for the male and female populations. The male vs. female MD and SMD (with 95% Cis) were also compared. A random‐effects model was applied due to the anticipated heterogeneity among studies, and the results are presented in forest plot format. For the proportions (pooled prevalence and pooled visualization rate) estimated to be less than 10% or more than 90%, the standard error of the mean and 95% CIs were calculated through the binomial exact method rather than the normal approximation method. Statistical heterogeneity was assessed using Cochran's *Q* test and quantified by the *I* ^2^ statistic. A Cochran's *Q* test *p*‐value < 0.10 indicated significant heterogeneity between studies. The Higgins *I* ^2^ statistic was defined as follows: 0%–40% as “might not be important,” 30%–60% as “moderate heterogeneity,” 50%–90% as “substantial heterogeneity,” and 75%–100% as “may represent considerable heterogeneity” [27].

Publication bias was evaluated for the primary outcomes by non‐parametric Begg's test, parametric Egger's regression, and the trim‐ and ‐fill method. However, funnel plot asymmetry was not applicable due to the < 10 number of studies for each primary outcome. Before publication bias assessment, Logit data transformation was utilized in case of non‐normal distribution of proportional outcomes (pooled prevalence and pooled visualization rate). The significance level was set at a *p*‐value < 0.05 except for the Begg's test, where a *p*‐value < 0.10 was considered statistically significant.

## 3 Results

### 3.1 Study Selection

**FIGURE 3:**
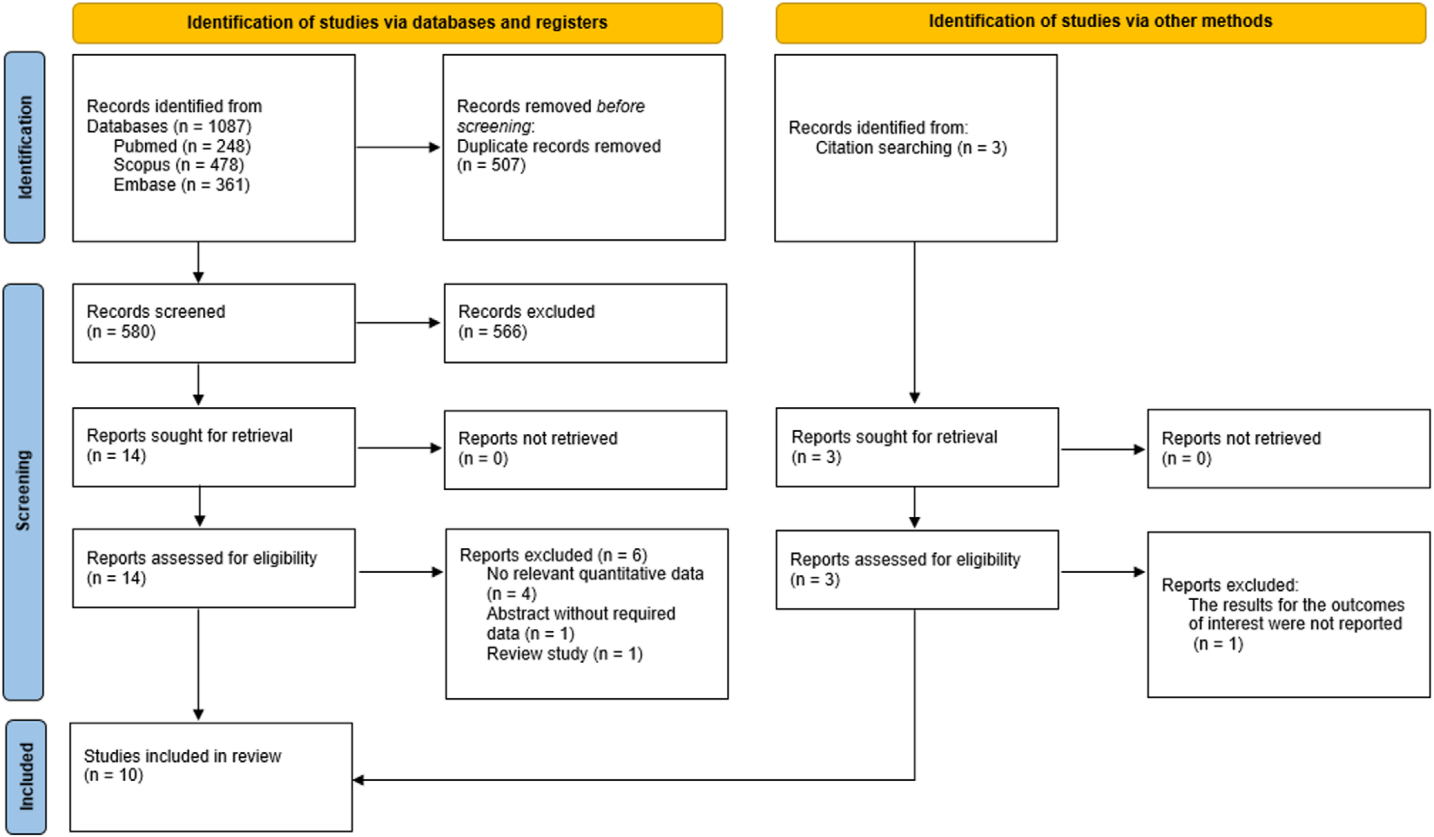
Flow diagram of the study identification and selection process, following Preferred Reporting Items for Systematic Reviews and Meta‐Analyses (PRISMA) guidelines.

A systematic search through the three online databases identified 1087 records, of which 580 underwent title and abstract screening after duplicate removal. Fourteen relevant articles were evaluated meticulously by their full texts; eight matched the inclusion criteria, and six were excluded for the reasons outlined in Figure 1. The citation tracking search yielded another three potential records; one was excluded, and two were eligible. Finally, 10 studies were included in this systematic review and meta‐analysis [24, 28, 29, 30, 31, 32, 33, 34, 35, 36]; all were eligible for data synthesis (Figure 3).

### 3.2 Study Characteristics

This review included ten studies published between 1997 and 2024; five studies were conducted in South Asia (China/Japan) [28, 29, 32, 34, 36], two in Turkey [24, 35], and one each in the UK [31], Italy [33], and Iran [30]. The sample size varied from 12 to 188 patients; the total number of participants was 597 (1194 hemi faces), of which 317 (53.1%) were female and 280 (46.9%) were male. The patients' age ranged from 18 to 77 years old, with a mean age of 34.85. BMI values were only available for 160 patients [24, 30, 32], the mean of which was 26.15 ± 6.46 kg/m^2^ (Table 1). Detailed anatomical data of the FA reported by each included study are displayed in Table 2.

**TABLE 1:** Baseline demographic characteristics of each included study in this meta‐analysis.

First author	Publication year	Continent	Country	Number of patients	Number of he‐mi‐faces	Number of males	Number of females	Age range	Mean age (SD)	BMI range (kg/m^2^)	Mean BMI (SD) (kg/m^2^)
Boz	2024	Europe/Asia	Turkey	188	376	94	94	18–60	39.3 (12.8)		
Pistoia	2023	Europe	Italy	41	82	20	21	26–61	32.0 (11.0)		
Shen	2023	Asia	China	33	66	13	20	20–49	27.0 (1.4)		22.3 (3.1)
Khorasanizadeh	2023	Asia	Iran	43	86	12	31	23–54	36.0 (8.3)	16.81–35.34	24.9 (4.1)
Ten	2020	Europe/Asia	Turkey	84	168	40	44	19–77	42.2 (11.9)	16.78–62.49	28.3 (7.5)
Renshaw	2007	Europe	UK	100	200	41	59	20–57	32.3		
Zhao	2002	Asia	Japan‐China	46	92	25	21	22–49	27.0 (6.8)		
Ariji	2001	Asia	Japan‐China	38	76	20	18	22–29	24.1 (1.8)		
Zhao	2000	Asia	China	12	24	8	4	18–57	28.5 (10.4)		
Nagase	1997	Asia	Japan	12	24	7	5	22–50	32.8 (7.1)		

Abbreviation: BMI, body mass index.

**TABLE 2:** Summarized data of each included study in this meta‐analysis.

First author	Publication year	Number of patients	Number of he‐mi‐faces	At the level of the lower border of the mandible (level 1)	At the level of Cheilion (level 2)	At the level of the lateral nasal ala (level 3)	FA course variation according to NLF
Boz	2024	188	376				Type A:198 (R:99, L:99) Type B:24 (R:13, L:11) Type C:32 (R:14, L:18) Type D: 122 (R:62, L:60)
Pistoia	2023	41	82	Visualized FA: 82 (R:41, L:41) Mean Depth: 6.6 (1.8)	Visualized FA: 80 (R:41, L:39) Mean Depth: 6.2 (1.45)	Visualized FA: 78 (R:41, L:37) Mean Depth: 4.44 (1.06)	
Shen	2023	33	66	Visualized FA: 66 (R:33, L:33) Mean Depth: 4.45 (1.34) Mean flow diameter: 1.56 (0.36)	Visualized FA: 66 (R:33, L:33) Mean Depth: 6.43 (1.14) Mean flow diameter: 1.4 (0.37)	Visualized FA: 66 (R:33, L:33) Mean Depth: 4.93 (1.57) Mean flow diameter: 1.32 (0.34)	Type A:33 (R:16, L:17) Type B:8 (R:4, L:4) Type C:21 (R:11, L:10) Type D: 4 (R: 2, L:2)
Khorasanizadeh	2023	43	86	Visualized FA: 86 Mean Depth: 7.76 (2.08)	Visualized FA: 83 Mean Depth: 10.4 (1.61)	Visualized FA: 77 Mean Depth: 12.8 (2.33)	
Ten	2020	84	168	Visualized FA: 168 (R:84, L:84) Mean Depth: 6.3 (2.12) Mean flow diameter: 1.56 (0.35)	Visualized FA: 168 (R:84, L:84) Mean Depth: 8.78 (2.78)	Visualized FA: 168 (R:84, L:84) Mean Depth: 10.02 (3.27)	Type A:61 (R:30, L:31) Type B:31 (R:15, L:16) Type C:48 (R:21, L:27) Type D: 28 (R:18, L:10)
Renshaw	2007	100	200	Visualized FA: 199 Mean flow diameter: 2.6 (0.45)	Mean flow diameter: 2.1 (0.41)	Mean flow diameter: 1.8 (0.39)	
Zhao	2002	46	92	Visualized FA: 92 (R:46, L:46) Mean flow diameter: 2.2 (0.6)	Visualized FA: 85 (R:43, L:42)		
Ariji	2001	38	76	Visualized FA: 76 (R:38, L:38) Mean flow diameter: 1.8 (0.4)			
Zhao	2000	12	24	Visualized FA: 24 (R:12, L:12) Mean flow diameter: 2.6 (0.5)	Visualized FA: 24 (R:12, L:12) Mean flow diameter: 1.9 (0.4)	Visualized FA: 22 (R:12, L:10) Mean flow diameter: 1.6 (0.3)	
Nagase	1997	12	24	Visualized FA: 24 (R:12, L:12) Mean flow diameter: 2.72 (0.8)	Visualized FA: 23 (R:12, L:11) Mean flow diameter: 1.87 (0.69)		

Abbreviations: FA, facial artery; NLF, nasolabial fold.

### 3.3 Risk of Bias Assessment

The application of the AQUA tool (developed for the quality assessment of anatomical studies included in meta‐analyses and systematic reviews) revealed that most of the studies (eight) had a “low” risk of bias in domain 1 (aim and subject characteristics), though two [28, 29] had a “high” risk of bias since they had not clearly defined the patients' baseline health status (question 3). All studies showed a “low” risk of bias in domain 2 (study design) as they utilized Doppler US, a widely accepted method for FA anatomical identification. Regarding domain 3 (methodology characterization), six studies were “high” risk due to the lack of clearly stating the specialty or the experience of the sonographic examiners (question 11). Besides, two studies [28, 29] provided insufficient details about their Doppler procedures and measures to eliminate inter‐observer and intra‐observer variability (question 13), leading to limited reproducibility (question 10). Concerning domain 4 (descriptive anatomy), all studies presented a “low” risk of bias except one [16] that had not precisely defined its “distance” parameter (question 17). In domain 5 (results reporting), four studies had a “low” risk of bias. In contrast, six were labeled as “high” risk, of which five had not mentioned any potential confounders such as sex or BMI (question 24), and two [30, 31] presented the results only in total status, meaning the data were not separately described for each hemi face (question 22). Only two recent studies [24, 32] had “low” bias risk across all five AQUA tool domains. The details of the risk of bias assessment by the AQUA application are summarized in Table 3.

**TABLE 3:** Details of the risk of bias assessment by the AQUA tool.

First author	Publication year	Domain 1: Aim and subject characteristics				Domain 2: Study design					Domain 3: Methodology characterization						Domain 4: Descriptive anatomy					Domain 5: Results reporting				
		1	2	3	4*	5	6	7	8	9*	10	11	12	13	14	15*	16	17	18	19	20*	21	22	23	24	25*
Boz	2024	Y	Y	Y	Low	Y	Y	Y	Y	Low	Y	N	Y	Y	Y	High	Y	Y	Y	Y	Low	Y	Y	Y	Y	Low
Pistoia	2023	Y	Y	Y	Low	Y	Y	Y	Y	Low	Y	Y	Y	Y	Y	Low	Y	Y	Y	Y	Low	Y	Y	Y	N	High
Shen	2023	Y	Y	Y	Low	Y	Y	Y	Y	Low	Y	Y	Y	Y	Y	Low	Y	Y	Y	Y	Low	Y	Y	Y	Y	Low
Khorasanizadeh	2023	Y	Y	Y	Low	Y	Y	Y	Y	Low	Y	Y	Y	Y	Y	Low	Y	N	Y	Y	High	Y	N	Y	Y	High
Ten	2020	Y	Y	Y	Low	Y	Y	Y	Y	Low	Y	Y	Y	Y	Y	Low	Y	Y	Y	Y	Low	Y	Y	Y	Y	Low
Renshaw	2007	Y	Y	Y	Low	Y	Y	Y	Y	Low	Y	N	Y	Y	Y	High	Y	Y	Y	Y	Low	Y	N	Y	N	High
Zhao	2002	Y	Y	Y	Low	Y	Y	Y	Y	Low	Y	N	Y	Y	Y	High	Y	Y	Y	Y	Low	Y	Y	Y	N	High
Ariji	2001	Y	Y	Y	Low	Y	Y	Y	Y	Low	Y	N	Y	Y	Y	High	Y	Y	Y	Y	Low	Y	Y	Y	Y	Low
Zhao	2000	Y	Y	N	High	Y	Y	Y	Y	Low	N	N	Y	U	Y	High	Y	Y	Y	Y	Low	Y	Y	Y	N	High
Nagase	1997	Y	Y	N	High	Y	Y	Y	Y	Low	N	N	Y	U	Y	High	Y	Y	Y	Y	Low	Y	Y	Y	N	High

*Note:* Y, Yes. N, No. U, Unclear. The initial signaling questions in each domain are answered as “Yes”, “No”, or “Unclear,” indicative of low, high, and unclear risk of bias, respectively. * indicates a risk‐of‐bias question. The ending question in each domain is a risk‐of‐bias question, judged as “Low”, “High”, or “Unclear. If all signaling questions for a domain were answered” Yes “then the risk of bias was judged” Low “If any signaling question was answered”, No “it indicated the potential for bias and was labeled as” High “risk of bias”. **Domain 1**, 1. Was the aim of the study clearly defined? 2. Was the chosen patient sample(s) appropriate for the aim of the study? 3. Are the baseline and demographic characteristics of the patient (age, sex, healthy or diseased, etc.) appropriate and clearly defined? 4. Could the method of patient selection have in any way introduced bias into the study? **Domain 2**, 5. Was the study design appropriate for the given aim of the study and address the research question(s)? 6. Were the materials used in the study appropriate for the given aim? 7. Were the methods used in the study appropriate for the given aim of the study? 8. Was the study design, including methods/techniques applied in the study, widely accepted or standard in the literature? 9. Could the study design have introduced bias into the study? **Domain 3**, 10. Are the methods/techniques applied in the study described with sufficient details to allow for reproducibility? 11. Was the specialty or the experience of the individual(s) performing the cadaveric dissection, assessing the imaging, or performing the study clearly stated? 12. Are details regarding the materials and tools/equipment (dissection microscopes, imaging machines, etc.) used in the study presented? 13. Was the methodology of the study indicative of measures taken to eliminate inter‐observer and intra‐observer variability, where relevant? 14. Do the images presented in the study indicate an accurate implementation of the methods/techniques (imaging, cadaveric, intraoperative, etc.) applied in the study? 15. Could the modality and methodology have introduced bias into the study? **Domain 4**, 16. Was the definition(s) of the reference standard(s) (i.e., normal or developmental anatomy) accurately and described in the study? 17. Were the outcomes and parameters (i.e., variation, anomaly, or abnormality) assessed in the study appropriate and clearly defined? 18. Were the figures (i.e., images, illustrations, diagrams, etc.) presented in the study clear and understandable? 19. Were any ambiguous anatomical observations (i.e., those prone to be classified as “others”) clearly described/depicted? 20. Could the descriptive anatomy have introduced bias into the study? **Domain 5**, 21. Was the statistical analysis performed appropriate and precise? 22. Are the reported results presented in the study clear and comprehensible, and were reported values consistent throughout the manuscript? 23. Do the reported numbers or results always correspond to the number of subjects in the study? If not, do the authors clearly explain the reason(s) for patient exclusion? 24. Are all potential confounders identified in the study? If so, are the confounders reported or, if needed, measured, and evaluated? 25. Could the reporting of the results have introduced bias into the study?

## 4 Meta‐Analysis Results

### 4.1 Co‐Primary Outcomes

#### 4.1.1 Facial Artery Visualization Rate

*Level 1 (lower border of mandible)*: The overall analysis of nine studies [24, 28, 29, 30, 31, 32, 33, 34, 36] (409 patients, 818 hemi faces) revealed a pooled visualization rate of 100.0% (95% CI: 99.9%–100.1%) regarding the FA at level 1, with no observation of heterogeneity among studies (*I* ^2^: 0.000%, *p* = 0.998). The FAs visualized on the right and left sides both had a pooled prevalence of 100.0% (95% CI: 99.8%–100.2%) with no heterogeneity among seven [24, 28, 29, 32, 33, 34, 36] (266 patients, 532 hemi faces) studies (*I* ^2^: 0.000%, *p* = 1.000). The bilateral visualization of FAs at the lower border of the mandible in the same seven studies showed a pooled prevalence of 100.0% (95% CI: 99.8%–100.2%), again with no heterogeneity (*I* ^2^: 0.000%, *p* = 1.000). In other words, all 266 FAs in both right and left hemi faces were detected by US at level 1 in 266 patients.

*Level 2 (cheilion)*: There were seven studies [24, 28, 29, 30, 32, 33, 36] (271 patients, 542 hemi faces) with an available number of Doppler‐detected FAs at level 2, of which six [24, 28, 29, 32, 33, 36] (228 patients, 456 hemi faces) reported the numbers separately for each side. The total detection rate at level 2 was 99.9% (95% CI: 99.5%–100.3%) with substantial heterogeneity (*I* ^2^: 56.369, *p* = 0.033). The right and left hemi faces demonstrated a pooled visualization rate of 100.0% (95% CI: 99.8%–100.2%) (*I* ^2^: 0.000%, *p* = 0.668) and 99.9% (95% CI: 99.4%–100.5%) (*I* ^2^: 33.869%, *p* = 0.182), respectively. Exactly 179 out of 182 patients demonstrated a bilateral detection of FAs in both hemi faces by Doppler US, presenting a pooled bilateral FA visualization rate of 100.0% (95% CI: 99.8%–100.2%) with nil heterogeneity (*I* ^2^: 0.000%, *p* = 0.527).

*Level 3 (lateral nasal ala)*: The FAs visualized at the level of the lateral nasal ala demonstrated a pooled prevalence of 99.8% (95% CI: 99.3%–100.4%) among five studies [24, 29, 30, 32, 33] (213 patients, 426 hemi faces). Given the *I* ^2^ of 75.648 and Cochran's *p*‐value of 0.002, this data may have considerable heterogeneity. The right and left hemi faces had a pooled visualization rate of 100.0% (95% CI: 99.8%–100.2%) (*I* ^2^: 0.000%, *p* = 1.000) and 99.9% (95% CI: 99.2%–100.6%) (*I* ^2^: 56.064%, *p* = 0.078) in four studies [24, 29, 32, 33] (170 patients, 340 hemi faces), respectively. Regarding the bilateral detection of FA at level 3, 164 out of 170 patients had FAs visible by Doppler US on both sides, leading to a 99.9% (95% CI: 99.2%–100.6%) pooled visualization rate (*I* ^2^: 0.000%, *p* = 1.000). These results are demonstrated in Table 4.

**TABLE 4:** Statistical results of meta‐analysis, the pooled Doppler visualization rate of the facial artery at three anatomical landmarks (co‐primary outcome).

Anatomical landmark	Number of studies	Number of patients	Number of he‐mi‐faces	Number of visualized facial arteries	Pooled visualization rate (%)	95% CI (%)	*I* ^2^ value (%)	Cochran's *Q* test *p*‐value
**The lower border of the mandible (level 1)**								
Total	9	409	818	817	100.0	99.9–100.1	0.000	0.998
Right	7	266	532	266	100.0	99.8–100.2	0.000	1.000
Left	7	266	532	266	100.0	99.8–100.2	0.000	1.000
Bilateral	7	266	532	266*2 = 532	100.0	99.8–100.2	0.000	1.000
**Cheilion (level 2)**								
Total	7	271	542	529	99.9	99.5–100.3	56.369	0.033
Right	6	228	456	225	100.0	99.8–100.2	0.000	0.668
Left	6	228	456	221	99.9	99.4–100.5	33.869	0.182
Bilateral	5	182	364	179*2 = 358	100.0	99.8–100.2	0.000	0.527
**Lateral nasal ala (level 3)**								
Total	5	213	426	411	99.8	99.3–100.4	75.648	0.002
Right	4	170	340	170	100.0	99.8–100.2	0.000	1.000
Left	4	170	340	164	99.9	99.2–100.6	56.064	0.078
Bilateral	4	170	340	164*2 = 328	99.9	99.2–100.6	56.064	0.078

Abbreviation: FA, facial artery.

#### 4.1.2 Facial Artery Course Variations According to the Nasolabial Fold

Only three studies [24, 32, 35] (305 patients, 610 hemi faces, 610 arteries) evaluated the relationship between the FA and the NLF. The most common type was type A (FA medial to NLF), with a pooled prevalence of 46.2% (95% CI: 34.9%–57.6%) in total, 45.7% (95% CI: 33.9%–57.5%) in the right, and 46.9% (95% CI: 35.8%–57.9%) in the left hemi face. In contrast, type B (FA lateral to NLF) was the least common course; its total pooled prevalence was 12.0% (95% CI: 3.8%–20.2%). The pooled prevalence of type C (FA crossing the NLF from medial to lateral) was 22.5% (95% CI: 5.9%–39.1%), posing type C as the second most common variation. Type D (FA crossing the NLF from lateral to medial) presented a pooled prevalence of 18.5% (95% CI: 2.9%–34.0%). Statistical heterogeneity was considerable in all four types as the *I* ^2^ was 84.932% in type A, 86.386% in type B, 95.037% in type C, and 96.033% in type D, along with *p*‐values below 0.10.

A symmetric course of the FA, according to the NLF, had a pooled prevalence of 29.0% (95% CI: 21.9%–36.0%), 6.5% (95% CI: 0%–13.4%), 8.6% (95% CI: 0%–18.4%), and 2.1% (95% CI: 0.4%–3.8%) for types A, B, C, and D, respectively. Thus, type A demonstrated the most symmetric course, while type D was the most asymmetric. Approximately half of the patients (pooled prevalence of 53.9% (95% CI: 45.9%–61.9%)) presented a symmetric course of FA according to the NLF, regardless of the type, with moderate heterogeneity (*I* ^2^: 40.628%, *p* = 0.186). The details of the analysis for FA course variations according to the NLF are shown in Table 5.

**TABLE 5:** Statistical results of meta‐analysis, the pooled prevalence of each facial artery course variation according to the nasolabial fold (co‐primary outcome).

The relationship between FA and NLF	Number of studies	Number of patients	Number of visualized facial arteries	Pooled prevalence (%)	95% CI (%)	*I* ^2^ value (%)	Cochran's *Q* test *p*‐value
**FA medial to NLF (Type A)**							
Total	3	305	610	46.2	34.9–57.6	84.932	0.001
Right	3	305	305	45.7	33.9–57.5	71.814	0.029
Left	3	305	305	46.9	35.8–57.9	67.956	0.044
Symmetry	3	305	610	29.0	21.9–36.0	37.942	0.200
**FA lateral to NLF (Type B)**							
Total	3	305	610	12.0	3.8–20.2	86.386	0.001
Right	3	305	305	11.6	4.2–19.0	67.019	0.048
Left	3	305	305	11.7	2.8–20.6	77.145	0.013
Symmetry	3	305	610	6.5	0–13.4	77.103	0.013
**FA crosses NLF from medial to lateral (Type C)**							
Total	3	305	610	22.5	5.9–39.1	95.037	0.000
Right	3	305	305	20.7	4.6–36.7	89.812	0.000
Left	3	305	305	23.2	5.7–40.7	90.513	0.000
Symmetry	3	305	610	8.6	0–18.4	85.047	0.001
**FA crosses NLF from lateral to medial** **(Type D)**							
Total	3	305	610	18.5	2.9–34.0	96.033	0.000
Right	3	305	305	20.3	4.3–36.3	91.998	0.000
Left	3	305	305	16.7	1.2–32.3	92.854	0.000
Symmetry	3	305	610	2.1	0.4–3.8	95.031	0.000
**Total symmetry**	3	305	610	53.9	45.9–61.9	40.628	0.186

Abbreviations: FA, facial artery; NLF, nasolabial fold.

### 4.2 Secondary Outcomes

#### 4.2.1 Facial Artery Mean Depth

There were four studies [24, 30, 32, 33] (201 patients, 402 hemi faces) concerning skin FA depth at the three anatomical levels and two [32, 33] (74 patients, 148 hemi faces) with available depth values exclusively for each side. The pooled mean skin‐FA depth at *level 1 (lower border of mandible)* was 6.27 mm (95% CI: 4.94–7.61 mm) for the total 402 arteries. At *level 2 (cheilion)*, 397 visualized FAs presented a pooled mean depth of 7.95 mm (95% CI: 5.97–9.93 mm). Regarding *level 3 (lateral nasal ala)*, the pooled mean depth was 8.04 mm (95% CI: 4.37–11.72 mm) among 389 arteries. These findings indicate that the FA gets deeper in the skin layers as it runs upper in the face from level 1 to level 3. However, the estimated total depths at all three levels had considerable heterogeneity (*I* ^2^: 98.2%, *p* < 0.001 at level 1, *I* ^2^: 99.3%, *p* < 0.001 at level 2, and *I* ^2^: 99.7%, *p* < 0.001 at level 3) (Table 6).

**TABLE 6:** Statistical results of meta‐analysis, the pooled mean depth of the facial artery in three anatomical landmarks.

Anatomical landmark	Number of studies	Number of patients	Number of visualized facial arteries	Pooled mean depth (mm)	95% CI (mm)	*I* ^2^ value (%)	Cochran's *Q* test *p*‐value
**The lower border of the mandible (level 1)**							
Total	4	201	402	6.27	4.94–7.61	98.2	< 0.001
Right	2	74	74	5.51	2.98–8.03	98.0	< 0.001
Left	2	74	74	5.53	3.85–7.22	95.5	< 0.001
**Cheilion (level 2)**							
Total	4	201	397	7.95	5.97–9.93	99.3	< 0.001
Right	2	74	74	6.17	5.86–6.47	0.0	0.702
Left	2	74	72	6.49	6.17–6.81	22.0	0.258
**Lateral nasal ala (level 3)**							
Total	4	201	389	8.04	4.37–11.72	99.7	< 0.001
Right	2	74	74	4.54	4.00–5.07	73.8	0.051
Left	2	74	70	4.74	4.36–5.12	21.6	0.259

Abbreviation: FA, facial artery.

No statistically significant difference was observed between the depths in the right and the left hemi faces at any level. However, there was a trend for deeper FAs in the left hemi face at levels 2 and 3 (Table S1).

#### 4.2.2 Facial Artery Mean Diameter

Seven studies [24, 28, 29, 31, 32, 34, 36] (325 patients, 650 hemi faces) assessed the diameter of total FAs at level 1, five [28, 29, 32, 34, 36] (141 patients, 282 hemi faces) assessed the diameter of FAs in each hemi face, and three [28, 29, 34] (62 patients, 124 hemi faces) assessed the diameter of total FAs in each gender (35 males, 27 females). The pooled mean FA diameter at *level 1 (lower border of mandible)* was 2.14 mm (95% CI: 1.74–2.54 mm) (649 arteries), 2.21 mm (95% CI: 1.85–2.58 mm) in 141 right‐sided, and 2.04 mm (95% CI: 1.68–2.41 mm) in 141 left‐sided FAs. The 70 and 54 FAs visualized at level 1 of 35 male and 27 female patients demonstrated a pooled mean diameter of 2.47 mm (95% CI: 1.81–3.12 mm) and 2.17 mm (95% CI: 1.59–2.75 mm), respectively. The male vs. female comparison demonstrated a 0.22 mm greater diameter in males at level 1 (MD 0.22 mm (95% CI: 0.00–0.44 mm), *I* ^2^: 0.0%, *p* = 0.943).

Three studies [28, 29, 32] (57 patients, 114 hemi faces) assessed the diameter of FAs at level 2 with available individual data for each hemi face. At *level 2 (cheilion)*, the total 113 detected FAs revealed a pooled mean diameter of 1.71 mm (95% CI: 1.33–2.10 mm), while 57 FAs observed on the right and 56 FAs on the left presented a pooled mean diameter of 1.75 mm (95% CI: 1.43–2.06 mm) and 1.63 mm (95% CI: 1.18–2.07 mm), respectively.

Two studies [29, 32] (45 patients, 90 hemi faces) assessed the diameter of FAs at level 3, with data reported for each side separately. Regarding *level 3 (lateral nasal ala)*, the pooled mean diameter was 1.46 mm (95% CI: 1.18–1.73 mm), 1.45 mm (95% CI: 1.35–1.55 mm), and 1.39 mm (95% CI: 1.04–1.74 mm) in 88 total, 45 right‐sided, and 43 left‐sided FAs, respectively. These findings indicate that the FA gets thinner from level 1 to level 3 as it branches out and approaches its termination point. However, the estimated total diameters at all three levels had considerable heterogeneity (*I* ^2^: 99.2%, *p* < 0.001 at level 1, *I* ^2^: 94.1%, *p* < 0.001 at level 2, and *I* ^2^: 92.5%, *p* < 0.001 at level 3) (Table 7).

**TABLE 7:** Statistical results of meta‐analysis, the pooled mean flow diameter of the facial artery in three anatomical landmarks.

Anatomical landmark	Number of studies	Number of patients	Number of visualized facial arteries	Pooled mean flow diameter (mm)	95% CI (mm)	*I* ^2^ value (%)	Cochran's *Q* test *p*‐value
**The lower border of the mandible (level 1)**							
Total	7	325	649	2.14	1.74–2.54	99.2	< 0.001
Right	5	141	141	2.21	1.85–2.58	95.5	< 0.001
Left	5	141	141	2.04	1.68–2.41	95.7	< 0.001
Male	3	35	70	2.47	1.81–3.12	96.0	< 0.001
Female	3	27	54	2.17	1.59–2.75	93.8	< 0.001
**Cheilion (level 2)**							
Total	3	57	113	1.71	1.33–2.10	94.1	< 0.001
Right	3	57	57	1.75	1.43–2.06	82.4	0.003
Left	3	57	56	1.63	1.18–2.07	90.3	< 0.001
**Lateral nasal ala (level 3)**							
Total	2	45	88	1.46	1.18–1.73	92.5	< 0.001
Right	2	45	45	1.45	1.35–1.55	0.0	0.320
Left	2	45	43	1.39	1.04–1.74	90.0	0.002

Abbreviation: FA, facial artery.

There was a statistically significant difference between the diameters in the right and the left hemi faces at level 2. The right‐sided FAs showed a 0.16 mm increased diameter (right vs. left MD 0.16 mm; 95% CI: 0.01–0.31 mm) compared with the left‐sided ones at the level of cheilion. Meanwhile, a trend for greater diameters in the right FAs vs. left FAs at levels 1 and 3 did not reach statistical significance (Table S2).

### 4.3 Tertiary Outcomes

#### 4.3.1 Facial Artery Terminative Branch

Two studies [24, 32] (117 patients, 234 hemi faces, 234 arteries) demonstrated that FA terminates with the AA in 71.8% (95% CI: 66.1%–77.5%) of cases in total, 71.8% (95% CI: 63.7%–80.0%) on the right, and 72.3% (95% CI: 64.5%–80.1%) on the left, indicating that AA is the most common terminative branch of the FA. FA termination on the LNA presented a pooled prevalence of 27.9% (95% CI: 22.2%–33.6%) in total, 27.4% (95% CI: 19.3%–35.4%) on the right, and 27.7% (95% CI: 19.9%–35.5%) on the left side. Thus, the LNA was the second most common final branch of the FA, followed by the AA. The SLA as the FA final branch showed a pooled prevalence of 5.7% (95% CI: 3.1%–8.4%) among 265 arteries (133 patients, 266 hemi faces) [31, 32]. No heterogeneity was retrieved between the studies, as the *I* ^2^ values were zero (Table S3).

#### 4.3.2 Facial Artery Branches Visualization Rate

The SLA and ILA were the most occurring branches of the FA with pooled visualization rates of 100% (95% CI: 99.7%–100.3% and 99.8%–100.2%, respectively) of the total 158 FAs [32, 36] (79 patients, 158 hemi faces). The AA was observed in 71.8% (95% CI: 66.1%–77.5%) of the 234 FAs visualized (117 patients, 234 hemi faces) [24, 32]. No statistical heterogeneity was observed between the studies (*I* ^2^: 0.000%).

### 4.4 Publication Bias

There was no evidence of publication bias regarding co‐primary outcomes among the included studies, except for the visualization rate of the FA at *level 1 (lower border of mandible)* reported by nine studies [24, 28, 29, 30, 31, 32, 33, 34, 36]. The Begg's test *p*‐value of 0.061 (*p* < 0.10) and Egger's of 0.000 (*p* < 0.05), along with an imputed study retrieved in the trim‐and‐fill method, suggest the publication bias regarding this data is inconsiderable since the incorporation of the imputed study did not change the effect size (Table S4).

## 5 Discussion

This is the first systematic review and meta‐analysis to comprehensively present FA anatomical data obtained via Doppler US. According to the results, US could detect FA in almost all cases at the three levels of the face. “FA running medial to the NLF” (type A) and “FA running lateral to the NLF” (type B) were the most and least frequent courses of the artery in relation to the NLF, respectively. Our data indicates that the FA gets deeper through the skin layers and narrower in diameter as it ascends the face and ramifies from level 1 to level 3. Larger diameters of FA were revealed in males vs. females at level 1 and on the right side vs. the left side at level 2. The FA termination on the AA was the most common ending pattern, while the SLA and ILA, detected in all cases, were the most occurring branches of the FA.

As the major blood supply of the anterior face [10], the FA sustains a high risk of vascular accidents during injections in its territory, such as NLF, superior/inferior lips, and nose augmentations [7, 24, 37]. One of the important complications following NLF augmentation is blindness, possibly due to anastomosis of AA, LNA, and DNA [2]. Thus, the morphometric knowledge of the FA developed in this study can provide an exemplary schema of pre‐procedural mapping for aestheticians to minimize the rate of intravascular injections during facial rejuvenating procedures.

Despite the robust knowledge of facial anatomy and years of practical expertise in traditional non‐guided injections by professional aestheticians, these blind injections still carry a serious risk of vascular events, as conventional methods for guiding these procedures, such as tactile feedback or facial landmarks, often fall short of precisely delineating complex facial anatomy and vasculature. This shortfall becomes even more critical when considering the inevitable individual anatomical and vascular variations, as well as distorted anatomy acquired after trauma, surgery, or non‐surgical procedures of the face [38]. Hence, pre‐procedural mapping by US is of great importance to improve the safety and precision of the cosmetic injections by individually customizing treatment plans [39]. According to evolving guidelines, three steps of Doppler US utilization within hyaluronic acid (HA) filler injections have been defined as follows [40].

(1) Pre‐procedural arterial mapping of only the targeted area to be filled (scan before injection, takes < 3 min) to provide insight about the position, path, and plane of the arteries to introduce the cannula or needle in a different plane [20]. Also, any previous fillings should be detected to avoid injections in the same place. This step helps to establish safe injection planning, addressing the patients' specific needs [41]. If no artery can be observed in this step, there is no need to perform the next steps. (2) Real‐time guidance of filler injection after inserting the cannula (scan while injection [20], takes 5–20 min): real‐time dynamic visualization of the needle/cannula tip allows for their correct positioning, particularly avoiding the plane where the arteries are located. (3) Immediate assessment of perfusion after filler injection (scan after injection, takes 1–3 min): to ensure the patency of adjacent arterial perfusion, sustained permeability of the vascularization of the filled area, and detection of early complications such as extrinsic vascular compression [22, 38, 40]. Furthermore, Doppler US also facilitates the diagnosis and treatment of ischemic complications by identification of turbulent flow in obstructed or compressed vessels, localization of errant or migrated filler material, and guiding the hyaluronidase injection [15, 23].

However, this three‐step technique is time‐consuming, difficult to learn, and needs simultaneous handling of the transducer and the syringe, which can be challenging. Therefore, a new time‐saving technique was proposed with a single step of Doppler US arterial mapping only in the area to be filled (pre‐injection mapping), rather than the entire face, without even marking on the patient's skin [42]. This is completely in line with our study's emphasis on the importance of the pre‐procedural vascular mapping step. Accordingly, a recent systematic review of 18 articles in this field declared that the majority of publications of interest concerned the application of US as a tool for vascular mapping, followed by its use for detection of previously injected fillers, which conveys the paramount role of step 1 in improving the procedure's safety [22].

### 5.1 Primary Outcomes

The visualization rates at three anatomical facial levels were measured to confirm the capability of Doppler US for FA detection. The results indicated that the US could be an efficient method for FA inspection since 100%, 99.9%, and 99.8% of all arteries were visualized at levels 1, 2, and 3, respectively (Table 4). However, the results regarding level 2 and level 3 demonstrated heterogeneities among studies, probably due to different techniques of the procedure [35] and the operator‐dependent nature of the US [33]. Accordingly, Lee et al. stated that Doppler US is qualified to detect nearly all arteries of the facial region before injection [43]. Therefore, it has been presented as a first‐line guidance for minimally invasive cosmetic procedures [22].

The NLFs are among the most common filler‐injected facial regions [2]. The FA ascends obliquely with varying tortuosity degrees within the NLFs' contiguity [7, 44]. Given this proximity and multiple variations in the FA course in relation to the NLF, this region is prone to vascular damage via injections [45]. Numerous studies by different modalities, including US, computed tomography angiography (CTA), and cadaveric dissection inquired about the various courses of the FA according to the NLF to implement a safe procedure for NLF augmentation [35]. Our meta‐analysis of 610 arteries demonstrated that “FA running medial to the NLF” (type A) (46.2%) followed by “FA crossing medial to lateral of the NLF” (type C) (22.5%) were the most frequent courses of FA in relation to NLF, whereas “FA running lateral to the NLF” (type B) (12.0%) was the scarcest variation (Table 5). This finding is completely in line with Ten et al. [24] who documented the same frequency order (A > C > D > B) of the four types of FA courses according to the NLF. On the contrary, Yang et al. [46] reported the frequency order of the four types in cadavers as follows: A (42.9%) > B (23.2%) > C (19.6%) > D (14.3%). Hence, the “FA course lateral to the NLF” (type B) demonstrated an almost two‐fold higher prevalence in cadavers than in Doppler‐detected arteries. Koziej et al. [47] declared the order of prevalence provided by CTA as follows: A (65.5%) > D (12.7%) > B (12.3%) > C (9.5%). Another CTA study of 300 FAs in 150 Asian individuals revealed the order of frequency as A (72.3%) > B (14.7%) > D (7.3%) > C (5.7%), declaring the highest reported prevalence for type A (72.3%) compared with other studies [48]. CTA, the first preoperative imaging choice for facial reconstructive surgeries [47], provides a more objective anatomical description than user‐dependent US. The most consistent finding of these comparisons is type A being the most frequent course of FA according to the NLF, with a higher prevalence in CTA (65.5%–72.3%) studies than cadaveric (42.9%) or Doppler studies (46.2%). Thus, greater caution should be considered during the injection of filler medially to the NLF since in 87.2% of all cases (three types of A, C, and D), the FA runs in the medial aspect of the NLF at least in a part of its course. The pooled incidence of adverse events following treatment of the NLF area is estimated to be 58%. Most transient and reversible complications include lumpiness, tenderness, swelling, bruising, pain, and redness. However, the pooled incidence of serious adverse events is 1% for infection and vascular events [49].

### 5.2 Secondary Outcomes

The depth of the FA determines the optimal length of the needle through the skin layers. Our data indicate that the FA gets more profound in the skin layers as it ascends in the face from level 1 (6.27 mm) to level 3 (8.04 mm) (Table 6). This pattern is mainly driven by Ten et al. [24] and Khorasanizade et al. [30], while Pistoia et al. [33] demonstrated a reverse pattern of the deeper artery at level 1 (~6 mm) and a more superficial artery at level 3 (~4 mm). The included study of Shen et al. [32] presented a superficial course of the FA at level 1 and level 3 (~4 mm) and a profound course at level 2 (~6.5 mm). Considering the estimated 6–8 mm range, more superficial injections than 6 mm or deeper than 8 mm can be more conservative options during lower face procedures between level 1 and level 3 [32]. However, given the mentioned discrepancies, no safe range for arterial depth can be defined by Doppler US to implement a specific instruction for needle trajectory [24]. The observed heterogeneity among studies can be attributed to the operator dependency of the US since the applied hand pressure can markedly affect the measured skin–FA depth. CTA is also incapable of arterial depth determination [47]. A cadaveric study by Lee et al. [50] confirmed our results by demonstrating the diversity and unpredictability of the FA depth. However, it suggested that the FA frequently runs subcutaneously over the facial muscles in the regions of the NLF [4, 45], inferolateral and superomedial aspects of the cheilion, and lateral nasal ala (equal to level 3) [50]. Therefore, the subcutaneous plane, probably the most high‐risk layer, should be avoided even though the best cosmetic advancements are achieved through injections to this layer [32]. Based on the anatomy delineated by cadaveric observations, a deep subperiosteal approach or intradermal injections through the skin layer are recommended for safer soft‐tissue filler depositions in the NLF area [4].

Our meta‐analysis exerted that the FA gets narrower in diameter from level 1 (2.14 mm) to level 3 (1.46 mm) as it branches out and approaches its termination point (Table 7). This declining trend of FA diameter from the lower border of the mandible to the lateral nasal ala level was consistent not only among the included studies [29, 31, 32] (Table 2) but also in cadavers (external diameter 1.9 ± 0.4 mm at level 1 to 1.1 ± 0.2 mm at level 3) [51]. The mean FA diameter at the inferior border of the mandible ranged between 1.46 ± 0.36 mm and 2.14 ± 0.38 mm by CTA in an Asian population, confirming our result at level 1 [48]. The widest range for FA diameter at level 1 was reported as 1.7–3.6 mm by previous literature from dissections, angiography, or Doppler US [10, 32]. Males presented almost 0.22 mm larger FA diameter than females at level 1, while the lack of data precluded us from carrying out the same comparison at levels 2 and 3. The right‐sided FAs were approximately 0.16 mm thicker than the left‐sided ones only at level 2 (Table S2), indicating that a greater blood supply is probably provided by the FA on the right side surrounding the perioral realm [32]. Knowing arterial diameter can be very practical as it helps the injectors choose the appropriate gauge of needle or cannula. Also, the increased diameter of FAs on the right side is valuable regarding right‐ vs. left‐sided dominancy.

There is a partial consensus that blunt‐tip cannulas are much more conservative than conventional bevel‐edged sharp needles since they are less capable of piercing the vessel walls [3, 50, 52, 53, 54]. However, the most appropriate and least hazardous gauge of needle/cannula has remained controversial. Several articles recommend small‐bore needles/cannulas since they are less traumatic [32, 45, 52, 55]. In contrast, some others preferred the large‐bore needles/cannulas (22G or 25 G) over the smaller ones (27G or 30 G) as they roll over the arteries [3] and let them slide away [53] instead of penetration [51]. Thus, regardless of the approach, the site‐specific awareness of arterial diameter helps determine the most appropriate device size for the targeted region.

### 5.3 Tertiary Outcomes

Ten et al. [24] reported the prevalence of AA as the final branch to be 72.7% on the right side and 78.6% on the left side, whereas Shen et al. [32] reported this as 59.1%. FA termination on the LNA had a prevalence of 16.7% in the right and 8.2% in the left hemi face, according to Ten et al. [24], and a total of 39.4% was reported by Shen et al. [32] The total prevalence of AA, LNA, and SLA as the final branch of the FA in our study was 71.8%, 27.9%, and 5.7%, respectively, indicating that AA, followed by LNA, is the most common terminative branches of the FA. The most up‐to‐date meta‐analysis [56] regarding FA termination patterns demonstrated a 69.81% prevalence for the summation of LNA and AA as the final branches among 2119 FAs under cadaveric dissections or CTAs, notably less than our estimate of 99.7% (AA 71.8% + LNA 27.9%). The measurements were specifically reported for AA and LNA as 30.40% and 43.83%, respectively. These comparisons indicate our probable overestimation of AA and underestimation of LNA prevalence as the final branches. These discrepancies can be mostly attributed to the differing visualization techniques (e.g., probe placement at the nose's proximity to detect LNA might be challenging via the US method).

Regarding FA branches, SLA and ILA were the most occurring branches detectable in all (100%) 158 cases. Accordingly, CTA observation of 255 FAs revealed that SLA (82.2%) followed by ILA (60.0%) were the most frequently presented branches of the FA [47]. The prevalence of SLA and ILA in cadaveric dissections was estimated as 77.5%–98.0% and 57.5%–100%, respectively [47], the two most common branches of the FA, again in line with our demonstration. In other words, the upper and lower lips stand as the most consistent territory of the FA.

Recently, growing evidence has advocated for the use of US in filler injections to increase safety, along with cosmetic outcomes and patient satisfaction, expanding its role as a valuable tool in aesthetic medicine. Thus, there is a paramount need to upgrade these recommendations to more standardized protocols and guidelines [39]. Accordingly, a four‐round Delphi consensus achieved a significant level of agreement among 15 international experts in US imaging from diverse specialties regarding the prerequisites of US application in cosmetic filler injection [13]. Similarly, recent literature has provided basic principles of US‐guided filler injection, including imaging optimization, required materials, operator position [57], and standard US transducer positions as well as injection techniques in different facial regions [58]. New handheld US with more affordable prices is emerging for more cost‐efficient scenarios [59].

Multifarious factors such as individual anatomical variation, movement artifacts, operators' skill, expertise, and specialty, technological diversity in US devices and transducer types all contribute to the formidable challenges of US imaging. AI incorporation into US technology can push these contemporary boundaries, representing a transformative approach in this field. AI algorithms are trained to improve the image quality by contrast enhancement and detail refinement, allowing for clearer visualization of critical anatomical structures. More importantly, AI enables real‐time automated analysis of US images and provides maps of facial anatomy; thereby assisting professionals to detect anatomical variation and abnormalities as well as vascular mapping with higher diagnostic accuracy and reduced operator variability. Hence, AI‐powered US holds great promise to revolutionize the aesthetic field by offering higher standardization, accuracy, safety, and personalized care [60].

### 5.4 Strengths and Limitations of the Study

This study is the first systematic review and meta‐analysis focusing on the various aspects of FA anatomy under Doppler US guidance. Doppler US can provide real‐time and functional anatomical knowledge of the vessels by detecting blood flow. Thus, the results are considered more dependable for the anatomical description of live individuals than those obtained from cadaveric observations, considering the anatomical distortion due to post‐mortem changes or fixation artifacts. Our corroboration of the data on different anatomical features with CTA and cadaveric evaluations confirms the results' reliability. Therefore, this study can be helpful to aestheticians in constructing a safe pre‐procedural injection plan, even though there are some limitations. *First*, some secondary and tertiary outcomes (but no primary) results mostly for side‐specific numbers (not total) were driven by merely a pair of available studies doubting the validity of the meta‐analysis results. However, the pooled meta‐analysis was still carried out to maintain the ordered structure of the context. Further prospective studies should be designed to address these knowledge gaps and provide more documentation for FA Doppler anatomy. *Second*, although the heterogeneity was substantial to considerable regarding some results, subgroup analysis was not applicable to detect possible sources of heterogeneity due to limitations in data. *Third*, the operator dependency of the US and, therefore, inter‐ and intra‐observer variability has always been problematic for this imaging method. *Fourth*, technical variations and differing baseline characteristics of targeted populations, notably BMI and race, may have contributed to inconsistency among the studies. Since the majority of included studies were from Asian countries, the meta‐analysis results may not fully reflect the global trend.

### 5.5 Future Perspectives

Future studies should include a larger sample size from around the world and diverse nationalities, particularly in domains that have been revealed to lack sufficient data in our current accumulation. The detailed documentation of patients' demographic characteristics, such as BMI, race, age, gender, previous clinical history (including aesthetic treatments or facial surgeries), technological details, and procedural techniques allows for possible subgroup analyses to diminish heterogeneity and reveal the sources of inconsistency among studies. These multi‐center, larger‐scale studies will pave the way for more standardization and the establishment of evidence‐based protocols and guidelines.

The reliability and efficiency of handheld devices might be explored to reduce expenses. The more advanced imaging technologies, such as contrast‐enhanced ultrasound (CEUS) and superb microvascular imaging (SMI), enhance the image quality, offering detailed vascular mapping, with better sensitivity for micro‐vessels and low‐flow detection. The convergence of US and AI must be explored as a potential revolutionary approach, since AI provides real‐time automated feedback and individualized data, supporting the decision‐making processes by superior diagnostic accuracy and safety.

In conclusion, Doppler US is capable of FA inspection in almost all cases at the three anatomical levels of the lower border of the mandible, cheilion, and lateral nasal ala. This systematic review and meta‐analysis study provides comprehensive anatomical knowledge of Doppler‐visualized FAs as an exemplary schema of pre‐procedural vascular mapping that can help aestheticians prevent intravascular injections, enhancing the safety of cosmetic procedures.

## Author Contributions

**Mohammad Reza Pourani:** conceptualization, data curation, methodology, writing – original draft, writing – review and editing. **Mandana Ebrahimzade:** data curation, formal analysis, validation, writing – original draft, writing – review and editing. **Ehsan Goudarzi:** formal analysis, validation, writing – original draft. **Martin Kassir:** supervision. **Reza Robati:** supervision. **Hamideh Moravvej:** supervision. **Fahimeh Abdollahimajd:** conceptualization, data curation, methodology, writing – original draft, supervision.

## Ethics Statement

Reviewed and approved by the Ethics Committee of Shahid Beheshti University of Medical Sciences, Tehran, Iran; IR.SBMU.SRC.REC.1403.020.

## Conflicts of Interest

The authors declare no conflicts of interest.

## Supporting information


**Table S1:** Facial artery skin depth difference between the right and left hemi face.
**Table S2:** Facial artery diameter difference between the right and left hemi face.
**Table S3:** Statistical results of meta‐analysis, the pooled prevalence of each facial artery termination pattern.
**Table S4:** Detailed publication bias assessment results for the co‐primary outcomes according to each test.

## Data Availability

The data that supports the findings of this study are available in the Data S1 of this article.
