# Supplementary material for: Evaluation of Facial Artery Course Variations, Diameters, and Depth Using Doppler Ultrasonography: A Systematic Review and Meta‐Analysis
Source: J Cosmet Dermatol. 2025 Aug 28;24(9):e70431. doi: 10.1111/jocd.70431 (PMC12392242; doi:10.1111/jocd.70431)
Supplement: Supplementary file 1 — Table S1: Facial artery skin depth difference between the right and left hemi face. Table S2: Facial artery diameter difference between the right and left hemi face. Table S3: Statistical results of meta‐analysis, the pooled prevalence of each facial artery termination pattern. Table S4: Detailed publication bias assessment results for the co‐primary outcomes according to each test. [file JOCD-24-e70431-s001.docx]

**Supplementary Table 1. Facial artery skin depth difference between the right and left hemi face**

| **Anatomical landmark** | **Right vs. Left depth MD (mm)** | **95% CI (mm)** | **I^2^ value (%)** | **Cochran's Q test p-value** | **Right vs. Left depth SMD (mm)** | **95% CI (mm)** | **I^2^ value (%)** | **Cochran's Q test p-value** |
| --- | --- | --- | --- | --- | --- | --- | --- | --- |
| **The lower border of the mandible (level 1)** | -0.06 | -0.90 - 0.78 | 64.1 | 0.095 | -0.05 | -0.61 - 0.51 | 65.7 | 0.088 |
| **Cheilion (level 2)** | -0.32 | -0.74 - 0.09 | 0.0 | 0.624 | -0.24 | -0.56 - 0.09 | 0.0 | 0.505 |
| **Lateral nasal ala (level 3)** | -0.26 | -0.66 - 0.14 | 0.0 | 0.760 | -0.20 | -0.53 - 0.13 | 0.0 | 0.584 |

**Supplementary Table 2. Facial artery diameter difference between the right and left hemi face**

| **Anatomical landmark** | **Right vs. Left diameter MD (mm)** | **95% CI (mm)** | **I^2^ value (%)** | **Cochran's Q test p-value** | **Right vs. Left diameter SMD (mm)** | **95% CI (mm)** | **I^2^ value (%)** | **Cochran's Q test p-value** |
| --- | --- | --- | --- | --- | --- | --- | --- | --- |
| **The lower border of the mandible (level 1)** | 0.11 | -0.05 - 0.28 | 54.1 | 0.069 | 0.27 | -0.11 - 0.65 | 56.7 | 0.055 |
| **Cheilion (level 2)** | **0.16** | **0.01 - 0.31** | **0.0** | 0.447 | **0.39** | **0.01 - 0.76** | **0.0** | 0.398 |
| **Lateral nasal ala (level 3)** | 0.10 | -0.14 - 0.34 | 59.7 | 0.115 | 0.31 | -0.43 - 1.05 | 58.2 | 0.122 |

**Supplementary Table 3. Statistical results of meta-analysis, the pooled prevalence of each facial artery termination pattern**

| **The final branch of FA** | **Number of studies** | **Number of patients** | **Number of visualized facial arteries** | **Pooled prevalence (%)** | **95% CI (%)** | **I^2^ value (%)** | **Cochran's Q test p-value** |
| --- | --- | --- | --- | --- | --- | --- | --- |
| **Angular artery (AA)** |  |  |  |  |  |  |  |
| Total | 2 | 117 | 234 | 71.8 | 66.1 - 77.5 | 0.000 |  |
| Right | 2 | 117 | 117 | 71.8 | 63.7 - 80.0 | 0.000 |  |
| Left | 2 | 117 | 117 | 72.3 | 64.5 - 80.1 | 0.000 |  |
| **Lateral nasal artery (LNA)** |  |  |  |  |  |  |  |
| Total | 2 | 117 | 234 | 27.9 | 22.2 - 33.6 | 0.000 |  |
| Right | 2 | 117 | 117 | 27.4 | 19.3 - 35.4 | 0.000 |  |
| Left | 2 | 117 | 117 | 27.7 | 19.9 - 35.5 | 0.000 |  |
| **Superior labile artery (SLA)** |  |  |  |  |  |  |  |
| Total | 2 | 133 | 265 | 5.7 | 3.1 - 8.4 | 0.000 |  |

FA, facial artery.

**Supplementary Table 4. Detailed publication bias assessment results for the co-primary outcomes according to each test**

| **Co-primary outcome** | **Number of studies** | **Begg's test**  **p-value** | **Egger's test**  **p-value** | **Trim-and-fill method number of imputed studies** |
| --- | --- | --- | --- | --- |
| **Pooled Doppler visualization rate of FA in three anatomical landmarks** |  |  |  |  |
| The lower border of the mandible (level 1) | 9 | **0.061** | **0.000** | **1** |
| Cheilion (level 2) | 7 | 1.000 | 0.071 | 0 |
| Lateral nasal ala (level 3) | 5 | 0.806 | 0.192 | 0 |
| **The pooled prevalence of each FA course variation according to NLF** |  |  |  |  |
| FA medial to NLF (Type A) | 3 | 1.000 | 0.709 | 0 |
| FA lateral to NLF (Type B) | 3 | 1.000 | 0.965 | 0 |
| FA crosses NLF from medial to lateral (Type C) | 3 | 1.000 | 0.829 | 0 |
| FA crosses NLF from lateral to medial (Type D) | 3 | 0.296 | 0.198 | 0 |

A **p-value < 0.10** was considered statistically significant in Begg's test, while for Egger's test**,** the significance level was set at **p-value < 0.05**
